# Arnold–Chiari malformation type I and the posterior dislocation of the odontoid process aggravate prolonged weaning in a patient with severe viral pneumonia: a case report

**DOI:** 10.1186/s12890-020-1078-1

**Published:** 2020-02-11

**Authors:** Renyu Ding, Yulan Meng, Xingjuan Jia, Xiaochun Ma

**Affiliations:** 1 Department of Intensive Care, The First Hospital of China Medical University, Nanjing Bei Street 155, Shenyang, 110001 Liaoning Province People’s Republic of China; 2 Department of Intensive Care, Tacheng Hospital of China Medical University, Tacheng, China

**Keywords:** Arnold-Chiari malformation, Prolonged weaning, Medulla oblongata compression

## Abstract

**Background:**

Prolonged and difficult weaning is associated with higher rates of complications and mortality. Therefore, it is important to identify the associated factors.

**Case presentation:**

We describe our experience with a 37-year-old man diagnosed with severe viral pneumonia (influenza A). He presented with acute respiratory failure type I on admission. During intubation, his blood pressure and heart rate decreased, and epinephrine and norepinephrine were administered. Although his clinical condition improved 8 days after intensive care unit (ICU) admission, he experienced difficulty weaning. He remained conscious but had a poor spontaneous cough with sputum production and weak limb muscle strength. His cough reflex was absent during bronchoscopic sputum suction, and he used abdominal breathing during the T-tube test. Magnetic resonance imaging revealed an Arnold–Chiari malformation type I, posterior dislocation of the odontoid process, and syringomyelia, with compression and deformation of the medulla and high cervical cord. The patient was successfully weaned from the ventilator at 20 days after ICU admission.

**Conclusions:**

Arnold–Chiari malformation type I and posterior dislocation of the odontoid process, which aggravate medullary compression and increase the risk of cervical nerve injury, might be a rare factor affecting prolonged weaning in critical illness.

## Background

Weaning from mechanical ventilation can be divided into three categories (simple, difficult, and prolonged) based on the difficulty and duration of the weaning process [1]. Patients with prolonged weaning are defined as those who have failed at least three weaning attempts or require more than 7 days of weaning [1]. Prolonged weaning is associated with higher rates of complications and mortality [2–4]. Many other factors can prolong weaning, including respiratory muscle weakness, abnormal respiratory mechanics, impaired gas exchange, cardiac dysfunction, and psychological distress. It is therefore important to identify the factors underlying a weaning difficulty [5, 6].

We herein report the interesting and unusual case of a patient with a difficult wean from ventilation following an acute viral chest infection, despite an improvement in chest status. Although the patient did not present with prominent signs and symptoms, he was found to have an Arnold–Chiari type I malformation with posterior dislocation of the odontoid process, which explained the prolonged weaning.

## Case presentation

A 37-year-old man was admitted to our intensive care unit (ICU) with a 3-day history of fever, cough, and respiratory distress and complaints of pain in the right side of the neck, numbness in the right upper limbs, and reduced pain sensation below the wrist of more than 6 months’ duration. He had no other significant medical history.

On admission, the patient had a body mass index of 24.6 kg/m^2^. He presented with acute respiratory failure (85% oxygen saturation on 5 L/min oxygen via mask), a temperature of 39.0 °C, blood pressure of 155/85 mmHg, pulse rate of 122 beats/minute, and respiratory rate of 35 breaths/minute. As hypoxemia persisted despite noninvasive ventilation (high-flow nasal cannula oxygen therapy), he was immediately intubated and ventilated. During intubation, his blood pressure and heart rate decreased, and epinephrine (1 mg single intravenous dose) and norepinephrine (0.7 μg/kg/min) were administered.

**Fig. 1:**
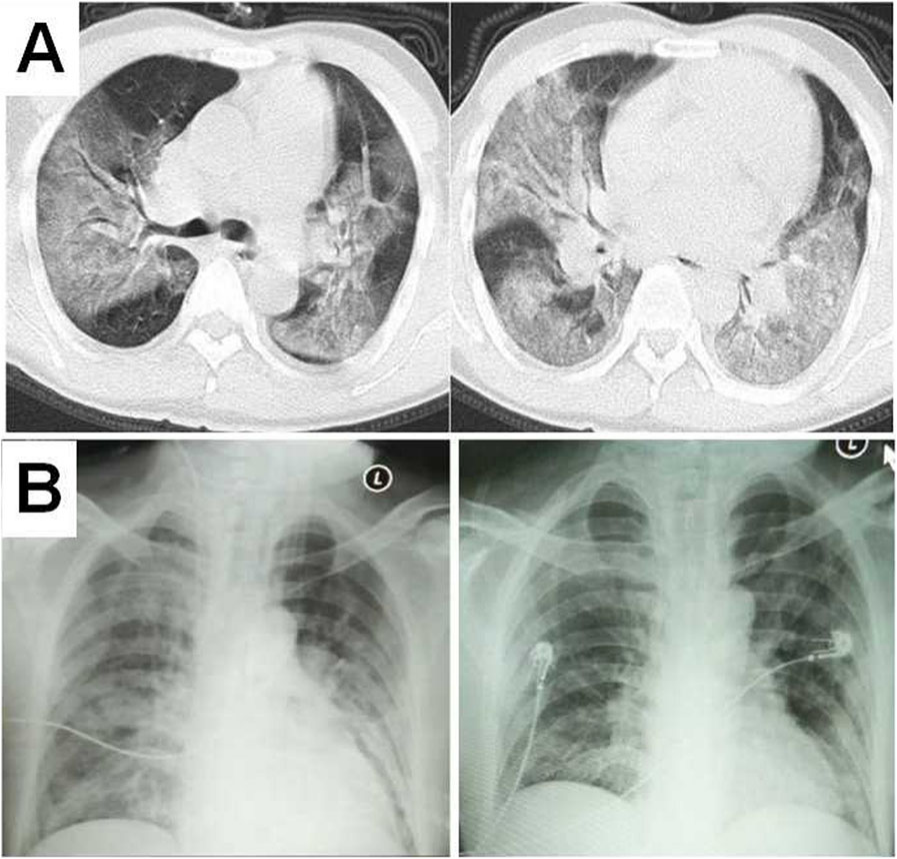
Computed tomography scan on admission to ICU showing extensive bilateral ground-glass opacities and consolidations(**a**). The chest X-ray examination on admission to ICU showing bilateral diffuse interstitial infiltrates (B, left). The chest X-ray examination on 8 days after admission to the ICU revealing the consolidative opacities in the left lower lung zones (**b**, right)

An arterial blood gas analysis revealed hypoxemia and hypocapnia (pH, 7.34; pCO_2_, 24.6 mmHg; pO_2_, 56 mmHg; HCO_3_, 12.8 mmol/L; base excess, − 11.1 mmol/L; lactate, 0.9 mmol/L on 5 L/min oxygen via mask). Chest computed tomography revealed extensive bilateral ground-glass opacities and consolidations (Fig. 1a). A chest X-ray examination showed bilateral diffuse interstitial infiltrates (Fig. 1b).

The following laboratory data were determined on admission: white blood cell count, 11,141 cells/μL (neutrophils, 87.9%; lymphocytes, 8.54%; and eosinophils, 1.3%); hemoglobin, 12.3 g/dL; platelet count, 264,000 cells/μL; international normalized ratio of prothrombin time, 1.51; aspartate aminotransferase, 120.5 IU/L; alanine aminotransferase, 53.6 IU/L; total bilirubin, 6.0 mg/dL; direct bilirubin, 2.6 mg/dL; total protein, 6.85 g/dL; albumin, 38.3 g/dL; urea nitrogen, 15.20 mmol/L; creatinine 124.7 μmol/L; C-reactive protein, 13.28 mg/dL; and procalcitonin, 8.16 ng/mL. A reverse transcription-polymerase chain reaction analysis of nasopharyngeal swab specimens collected in the emergency room on admission confirmed the patient had developed an influenza A (H3) infection.

Cefoperazone, sulbactam, moxifloxacin, and oseltamivir were administered for 8 days and methylprednisolone (40 mg bid) was given for 3 days. Eight days after admission to the ICU, the patient exhibited the following arterial blood gas findings: pH, 7.49; pCO_2_, 42.2 mmHg; pO_2_, 94 mmHg; HCO_3_, 31.5 mmol/L; base excess, 7.5 mmol/L; and lactate, 1.0 mmol/L (FiO_2_, 40%). Despite radiologically demonstrated improvements in his clinical condition and chest lesions (Fig. 1b), the patient experienced difficulty weaning. He remained conscious but had a poor spontaneous cough with sputum production and weak limb muscle strength (grade 2–3). His cough reflex was absent during bronchoscopic sputum suction, and he used abdominal breathing during the T-tube test. Two hours after the T-tube test, an arterial blood gas analysis revealed hypercapnia with a pH of 7.25, pCO_2_ of 94.2 mmHg, pO_2_ of 81 mmHg, and base excess of 10.3 mmol/L. These signs could not be easily attributed to the common causes of weaning difficulties (e.g., diaphragmatic dysfunction, ICU-acquired myasthenia). We therefore suspected a dysfunction in the respiratory center of the brain stem (e.g., brain stem infarct) and performed a CT examination of the brain. However, no anomalies were found.

**Fig. 2:**
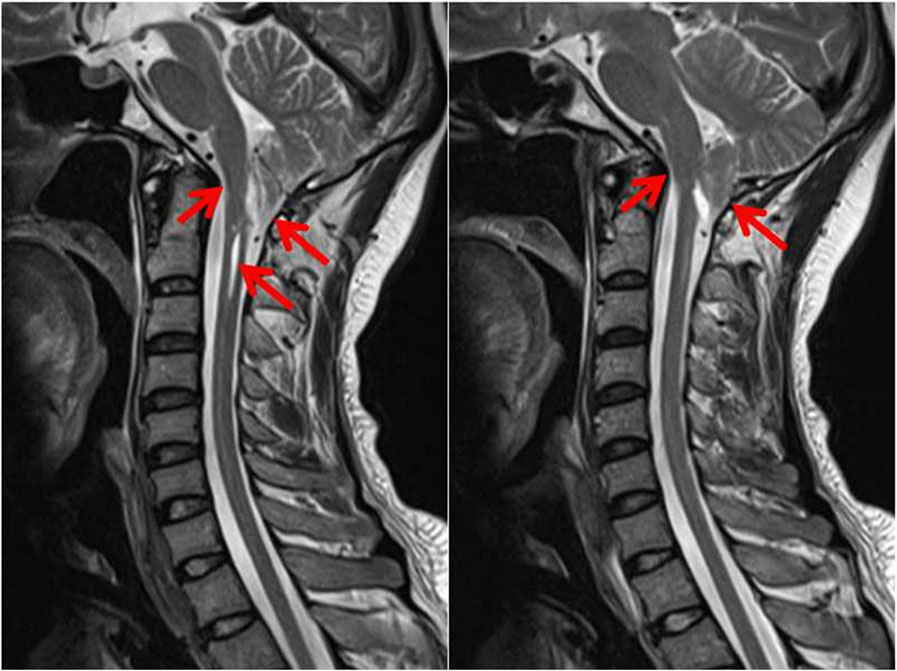
Magnetic resonance image of the cervical spine. Arrows indicate tonsillar herniation to the first cervical vertebra with a posterior dislocation of the odontoid process and syringomyelia. The medulla and high cervical cord are compressed and deformed

Given the patient’s weaning difficulty, a tracheotomy was performed, and sputum was drained under fiberoptic bronchoscopy at least twice per day. The patient was also administered early comprehensive rehabilitation, including psychological counseling. He successfully weaned from the ventilator at 20 days after admission to the ICU. Subsequent magnetic resonance imaging revealed a tonsillar herniation to the first cervical vertebra with a posterior dislocation of the odontoid process and syringomyelia, as well as compression and deformation of the medulla and high cervical cord (Fig. 2).

After weaning from the ventilator, the patient’s clinical condition rapidly improved. He was eventually discharged without undergoing surgery for the Arnold–Chiari malformation and posterior dislocation of the odontoid process. He had not experienced a recurrence of pneumonia, dysphagia, or respiratory failure after 2 months of follow-up. Although his cough and expectoration abilities were good, significant changes in his neurological symptoms and signs were apparent when compared with his status before pneumonia. For example, the patient occasionally experienced dizziness and visual rotation with postural changes and could not perform fine movements of the right hand, such as clasping and writing. Additionally, he continued to experience weak muscle strength in the right limbs (grade 4), numbness in the right upper limbs, and a reduced sensation of pain and temperature below the right wrist. The patient refused further surgical treatment for the Arnold–Chiari malformation and posterior dislocation of the odontoid process.

## Discussion and conclusions

The above report illustrates the diagnostic challenge faced when experiencing a case involving a weaning difficulty in a previously healthy young person and determining the possible underlying pathogenic mechanisms. In this case, an Arnold–Chiari malformation type I and posterior dislocation of the odontoid process aggravated the weaning process in a patient with severe viral pneumonia.

An Arnold–Chiari malformation, in which the cerebellar tonsils and vermis herniate below the level of the foramen magnum, is a relatively common condition caused by displacement of the brainstem medulla. Most cases of Arnold–Chiari malformation are designated as type I based on the caudal displacement of the cerebellar tonsils into the upper cervical spinal canal [7–9]. Although this type of herniation may be asymptomatic, a few previous studies have reported cases in which patients with an Arnold–Chiari malformation type I developed an acute type II respiratory failure [10–12]. Campisi et al. and Nathadwarawala et al. have reported that an adult with Arnold–Chiari malformation type I may initially present with recurrent aspiration, secondary bronchiectasis, dysphagia, or central sleep apnea [10, 11].

There are a number of possible mechanisms for type 2 respiratory failure in Chiari malformation, including alveolar hypoventilation secondary to diaphragmatic palsy and brainstem compression [12–14]. In contrast to most previously reported cases with type 2 respiratory failure, our patient initially presented with type I respiratory failure (pCO2, 24.6 mmHg) related to viral pneumonia, rather than Arnold–Chiari malformation. Our case was characterized by a weaning difficulty attributed to this Arnold–Chiari malformation and a posterior dislocation of the odontoid process. It is important to distinguish this type of weaning difficulty from phrenic nerve dysfunction or ICU-acquired myasthenia, as poor limb muscle strength or abdominal breathing are typical features of both conditions. In the present case, the main clinical manifestation supporting a diagnosis of compression of the respiratory center was the absence of a cough reflex during fiberoptic bronchoscopy suction. Moreover, this patient did not present with typical signs of medullary and high cervical cord compression before developing severe pneumonia. We note that this patient’s symptoms of neck pain, upper limb numbness, and reduced pain sensation below the wrist could also have been caused by cervical spondylosis. These features delayed and impeded the diagnosis.

In our present case, the explanation for prolonged weaning was complicated. Tonsillar herniation with posterior dislocation of the odontoid process might contributed to medullary compression, although this factor was mild. Medullary compression might have been aggravated by a long period of severe hypoxia, secondary brain edema, and elevated intracranial pressure, as well as by emergency endotracheal intubation while in the supine position. During intubation, the decreases in heart rate and blood pressure might have been caused by hypoxemia or compression of the medulla oblongata. The gradual removal of these possible factors led to relief of the medulla oblongata compression, recovery of the patient’s cough reflex, and successful weaning from the ventilator. However, the significant changes in neurological symptoms and signs during follow-up relative to those observed before pneumonia suggested a cervical nerve injury. Kijsirichareanchai et al. have reported a 49-year-old man developed acute hypercapneic respiratory failure during an episode of community-acquired pneumonia [12]. Similar with our case, the man was diagnosed with Chiari malformation type I after the hospitalization without typical neurological symptoms and signs. Suffering from the possible additional medullary and/or cervical cord injury related to the intubation, the patient required tracheostomy and prolonged mechanical ventilation (more than 1 year).

In conclusion, our present case illustrates a rare factor associated with prolonged weaning after a critical illness. Specifically, an Arnold–Chiari malformation and posterior dislocation of the odontoid process aggravated the medullary compression in this patient and increased the risk of cervical nerve injury during critical illness.

## Data Availability

All data and material analyzed during this study are included in this published article.

## Abbreviations

ACM: Arnold–chiari malformation
FiO2: Fraction of inspired oxygen
ICU: Intensive care unit
pCO2: Carbon dioxide partial pressure
pO2: Oxygen partial pressure
